# Supplementary material for: Effect of Sox18 on the Induction Ability of Dermal Papilla Cells in Hu Sheep
Source: Biology (Basel). 2022 Dec 30;12(1):65. doi: 10.3390/biology12010065 (PMC9855062; doi:10.3390/biology12010065)
Supplement: Supplementary file 1 [file biology-12-00065-s001.zip › biology-2059078-supplementary.pdf]

Supplementary Materials

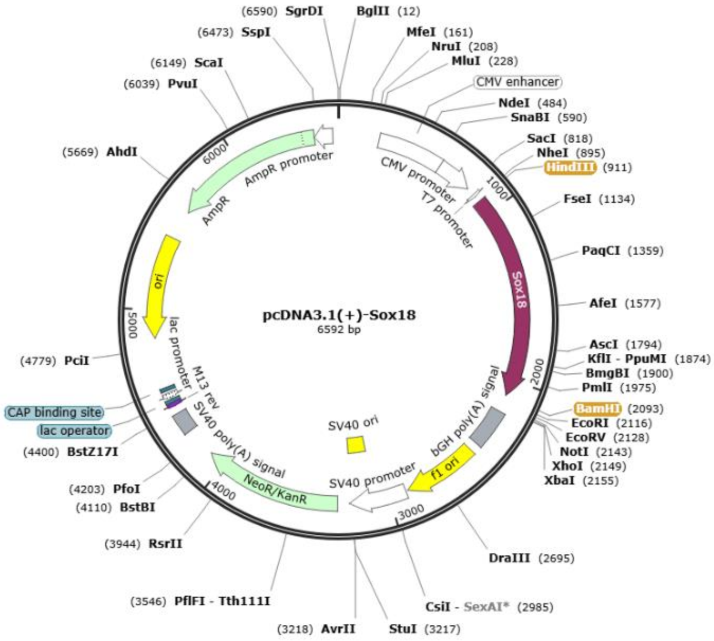

Figure S1. The pcDNA3.1(+)-*Sox18* plasmid illustration.

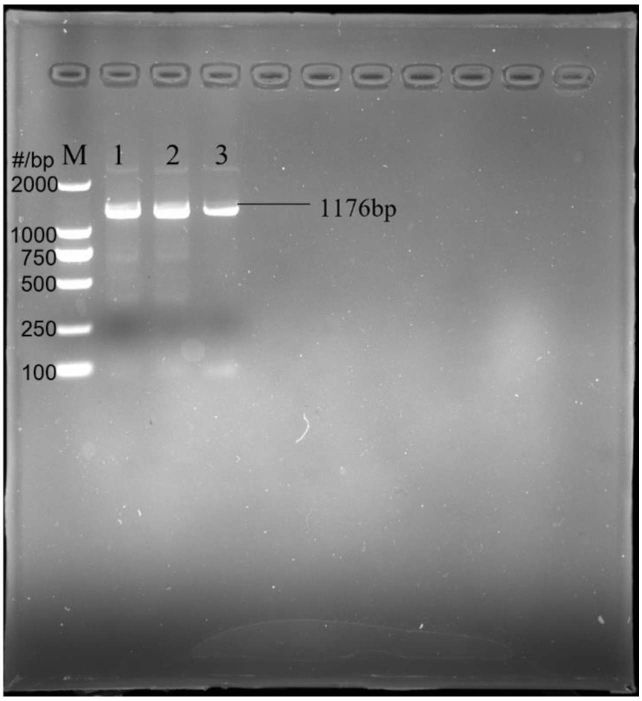

Figure S2. 1% agarose gel electrophoresis of amplification products of Hu sheep *Sox18* CDS (1176bp). Weight marker (molecular weight in bp): Takara QDL2,000 Quantitative DNA Marker, 100bp-2,000bp, Code No. 3580Q. M is QDL2,000 Quantitative DNA Marker. 1,2 and 3 are amplification products of Hu sheep *Sox18* CDS.

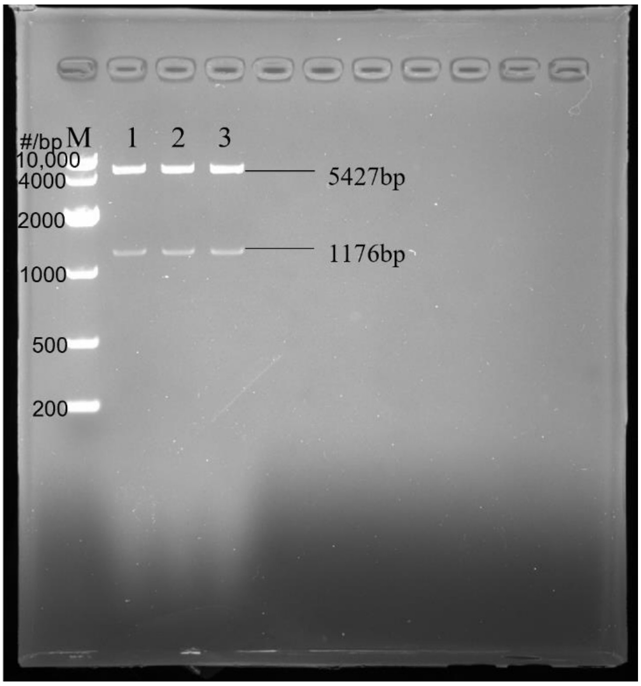

Figure S3. 1% agarose gel electrophoresis of double enzyme digestion products of pcDNA3.1(+)-*Sox18* (1176bp and 5427bp). Weight marker (molecular weight in bp): Takara DL10,000 DNA Marker, 200bp-10,000bp, Code No. 3584A. M is DL10,000 DNA marker. 1, 2 and 3 are double enzyme digestion products of pcDNA3.1(+)-*Sox18.*

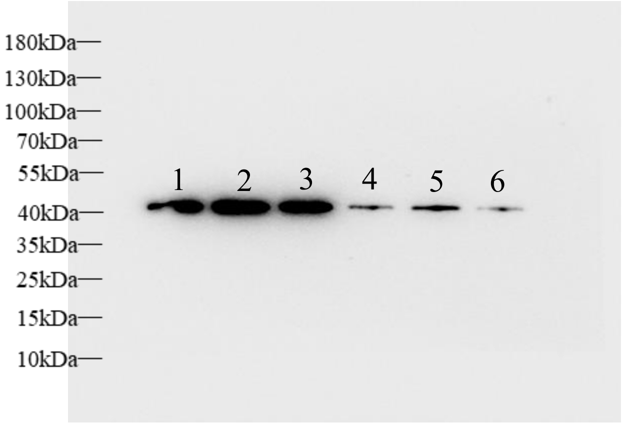

Figure S4. Western blot membrane of Sox18 (41kDa) protein from DPCs transfected with pcDNA3.1(+)-*Sox18* and pcDNA3.1(+). Weight marker (molecular weight in kDa): TSINGKE Trelief® Prestained Protein Ladder, 10kDa to 180kDa, catalogue number: TSP021. 1, 2, and 3 are protein of DPCs transfected with pcDNA3.1(+)-*Sox18*. 4, 5 and 6 are protein of DPCs transfected with pcDNA3.1(+).

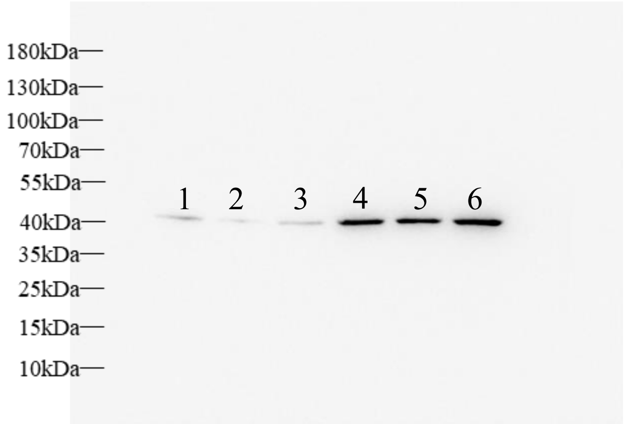

Figure S5. Western blot membrane of Sox18 (41kDa) protein from DPCs transfected with siRNA-*Sox18* and siRNA-NC. Weight marker (molecular weight in kDa): TSINGKE Trelief® Prestained Protein Ladder, 10kDa to 180kDa, catalogue number: TSP021. 1, 2, and 3 are protein of DPCs transfected with siRNA-*Sox18*. 4, 5 and 6 are protein of DPCs transfected with siRNA-NC.

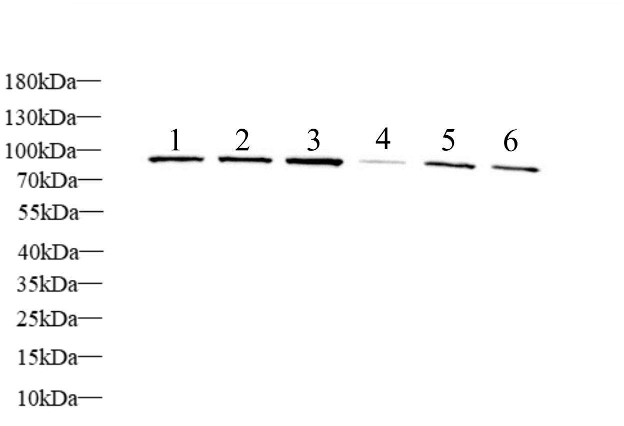

Figure S6. Western blot membrane of β-catenin (85kDa) protein from DPCs transfected with pcDNA3.1(+)-*Sox18* and pcDNA3.1(+). Weight marker (molecular weight in kDa): TSINGKE Trelief® Prestained Protein Ladder, 10kDa to 180kDa, catalogue number: TSP021. 1, 2, and 3 are protein of DPCs transfected with pcDNA3.1(+)-*Sox18*. 4, 5 and 6 are protein of DPCs transfected with pcDNA3.1(+).

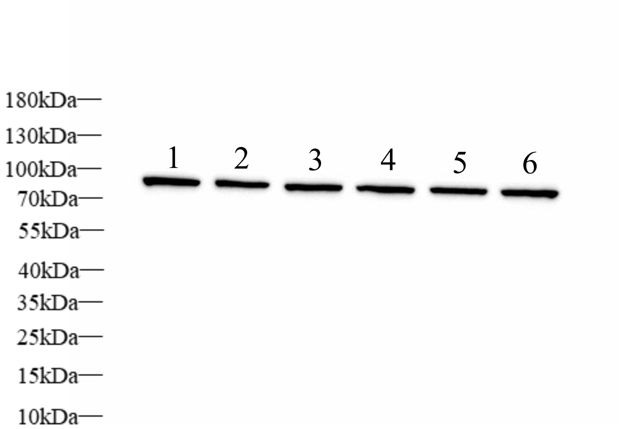

Figure S7. Western blot membrane of β-catenin (85kDa) protein from DPCs transfected with siRNA-*Sox18* and siRNA-NC. Weight marker (molecular weight in kDa): TSINGKE Trelief® Prestained Protein Ladder, 10kDa to 180kDa, catalogue number: TSP021. 1, 2, and 3 are protein of DPCs transfected with siRNA-*Sox18*. 4, 5 and 6 are protein of DPCs transfected with siRNA-NC.

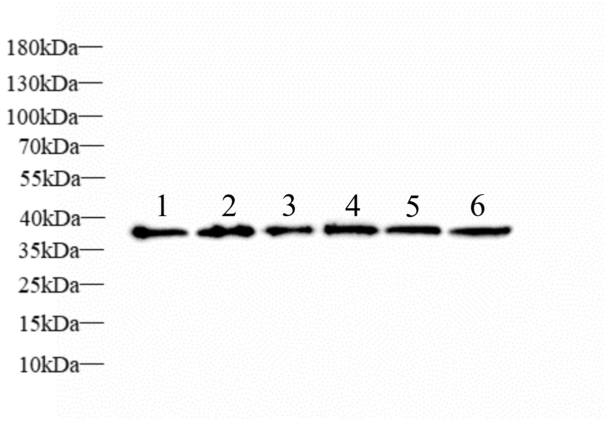

Figure S8. Western blot membrane of GAPDH (37kDa) protein from DPCs transfected with pcDNA3.1(+)-*Sox18* and pcDNA3.1(+). Weight marker (molecular weight in kDa): TSINGKE Trelief® Prestained Protein Ladder, 10kDa to 180kDa, catalogue number: TSP021. 1, 2, and 3 are protein of DPCs transfected with pcDNA3.1(+)-*Sox18*. 4, 5 and 6 are protein of DPCs transfected with pcDNA3.1(+).

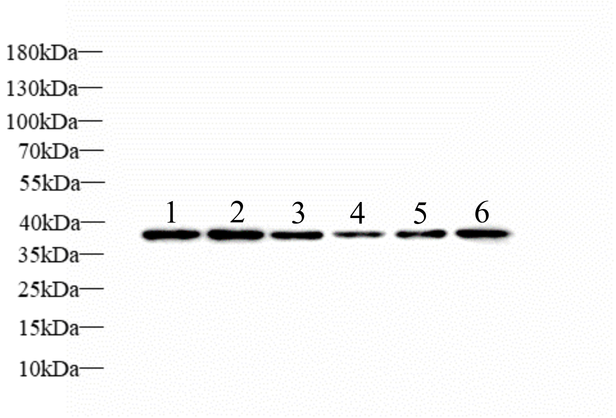

Figure S9. Western blot membrane of GAPDH (37kDa) protein from DPCs transfected with siRNA-*Sox18* and siRNA-NC. Weight marker (molecular weight in kDa): TSINGKE Trelief® Prestained Protein Ladder, 10kDa to 180kDa, catalogue number: TSP021. 1, 2, and 3 are protein of DPCs transfected with siRNA-*Sox18*. 4, 5 and 6 are protein of DPCs transfected with siRNA-NC.
